# Automatic Segmentation of the Infraorbital Canal in CBCT Images: Anatomical Structure Recognition Using Artificial Intelligence

**DOI:** 10.3390/diagnostics15131713

**Published:** 2025-07-04

**Authors:** Ismail Gumussoy, Emre Haylaz, Suayip Burak Duman, Fahrettin Kalabalık, Muhammet Can Eren, Seyda Say, Ozer Celik, Ibrahim Sevki Bayrakdar

**Affiliations:** 1 Department of Oral and Maxillofacial Radiology, Faculty of Dentistry, Sakarya University, Sakarya 54050, Turkey; 2 Department of Oral and Maxillofacial Radiology, Faculty of Dentistry, Inönü University, Malatya 44000, Turkey; 3 Department of Diagnostic Sciences, Texas A&M College of Dentistry, Dallas, TX 75226, USA; 4 Department of Mathematics Computer, Faculty of Science and Art, Eskişehir Osmangazi University, Eskişehir 26040, Turkey; 5 Department of Oral and Maxillofacial Radiology, Faculty of Dentistry, Eskişehir Osmangazi University, Eskişehir 26040, Turkey

**Keywords:** artificial intelligence, cone-beam computed tomography, deep learning, nnU-Net, infraorbital canal

## Abstract

**Background/Objectives:** The infraorbital canal (IOC) is a critical anatomical structure that passes through the anterior surface of the maxilla and opens at the infraorbital foramen, containing the infraorbital nerve, artery, and vein. Accurate localization of this canal in maxillofacial, dental implant, and orbital surgeries is of great importance to preventing nerve damage, reducing complications, and enabling successful surgical planning. The aim of this study is to perform automatic segmentation of the infraorbital canal in cone-beam computed tomography (CBCT) images using an artificial intelligence (AI)-based model. **Methods:** A total of 220 CBCT images of the IOC from 110 patients were labeled using the 3D Slicer software (version 4.10.2; MIT, Cambridge, MA, USA). The dataset was split into training, validation, and test sets at a ratio of 8:1:1. The nnU-Net v2 architecture was applied to the training and test datasets to predict and generate appropriate algorithm weight factors. The confusion matrix was used to check the accuracy and performance of the model. As a result of the test, the Dice Coefficient (DC), Intersection over the Union (IoU), F1-score, and 95% Hausdorff distance (95% HD) metrics were calculated. **Results:** By testing the model, the DC, IoU, F1-score, and 95% HD metric values were found to be 0.7792, 0.6402, 0.787, and 0.7661, respectively. According to the data obtained, the receiver operating characteristic (ROC) curve was drawn, and the AUC value under the curve was determined to be 0.91. **Conclusions:** Accurate identification and preservation of the IOC during surgical procedures are of critical importance to maintaining a patient’s functional and sensory integrity. The findings of this study demonstrated that the IOC can be detected with high precision and accuracy using an AI-based automatic segmentation method in CBCT images. This approach has significant potential to reduce surgical risks and to enhance the safety of critical anatomical structures.

## 1. Introduction

The infraorbital nerve passes through the inferior orbital fissure to enter the orbit and then travels along the infraorbital sulcus, which extends across the orbital floor. After this, it enters the infraorbital canal (IOC) and emerges through the infraorbital foramen, connecting to the surface structures. This nerve provides sensory innervation to the skin, muscles, and mucosa of the areas it passes through, including important structures such as the lower eyelid, the upper lip, and the gums [1,2,3].

The infraorbital nerve, a branch of the maxillary nerve, provides sensory innervation to the lower eyelid, the conjunctiva, the lateral wall of the nose, the nostrils, the upper lip, and the gums. This nerve is a key point for anesthesia in the infraorbital nerve block procedures commonly used in oral and maxillofacial surgery. The IOC and the foramen are of great importance during nerve block procedures. Therefore, knowing its exact localization is crucial to increasing the effectiveness of regional block anesthesia, thereby contributing to the safe and successful execution of surgical interventions [1,2,4].

Iatrogenic infraorbital nerve injury may occur during surgical procedures or fractures involving the anterior and superior walls of the maxilla. Surgical procedures such as rhinoplasties, Caldwell–Luc operations, tumor surgery, orbital floor reduction (blow-out fractures), malar region fractures, and Le Fort Type-I osteotomies are among those that may cause infraorbital nerve damage. Therefore, accurately knowing the anatomical location of the IOC is crucial to preventing iatrogenic infraorbital nerve injury during these surgical interventions [1,4,5,6].

Automatic segmentation is the process of digitally isolating anatomical structures (such as bones, blood vessels, nerves, organs, etc.) from medical imaging data [7,8,9,10]. This process allows for faster, more consistent, and more accurate results compared to those with manual segmentation. As a result, anatomical structures are analyzed more efficiently, providing significant advantages in treatment planning. Additionally, automatic segmentation can help create customized treatment plans based on each individual’s anatomical features. This leads to more effective and personalized diagnostic and treatment processes in healthcare [11,12].

Cone-beam computed tomography (CBCT) and computed tomography (CT) are the most commonly used methods for detailed imaging of bony structures such as the IOC. These techniques allow for clear visualization of the bony structures due to their high resolution [13,14,15]. Other imaging methods are effective in evaluating soft tissue and nerve structures [16,17,18]. Accurate segmentation of the IOC in CBCT images through automatic segmentation ensures safer and more effective surgical interventions while also increasing the success rate of anesthesia applications.

Some studies have been conducted on automatic segmentation of various anatomical structures in the maxillofacial region, such as the orbit, the maxillary sinus, and the nasal cavity [19,20,21]. However, there have been no studies regarding the automatic segmentation of the IOC, which is critical for certain surgical procedures in this region. Therefore, the aim of this study is to perform automatic segmentation of the IOC in CBCT images using the nnU-Net v2 model and to evaluate the model’s performance in IOC segmentation based on the metric values obtained.

## 2. Materials and Methods

### 2.1. The Study Design and Sample Size Criteria

In this study, CBCT images obtained from 110 patients who presented to the Department of Oral and Maxillofacial Radiology at the Faculty of Dentistry, Inönü University, were used. These images were acquired using the NewTom 5G device (Quantitative Radiology, Verona, Italy). The foundation of this study was the nnU-Net v2 model, which was employed for automatic segmentation of the IOC. All procedures were conducted in accordance with the ethical standards of the Declaration of Helsinki. The study protocol was approved by the Non-Interventional Clinical Research Ethics Committee of Inönü University (Approval No: 2025/7703).

The sample size for this study was determined using G*Power software (version 3.1.9.7; Franz Faul, Universitat Kiel, Kiel, Germany) with the alpha level (α) set at 0.05 and a desired statistical power of 95%. Under these conditions, it was calculated that including 110 samples would achieve a true study power of 95%.

In this study, CBCT images from 110 patients were used, and automatic segmentation was performed on a total of 220 IOCs. Of the individuals included in this study, 55 were male and 55 were female. The participants’ ages ranged from 18 to 64 years, with a mean age of 44. The inclusion criteria for this study were as follows:

- Individuals aged 18 years or older;
- Individuals who had not undergone any surgical procedures in the head and neck region;
- Individuals with no history of trauma or metabolic bone diseases;
- Patients with CBCT images in which the boundaries of the IOC could clearly be visualized.

The exclusion criteria for this study were as follows:

- CBCT images in which the boundaries of the IOC could not be clearly visualized;
- CBCT images containing artifacts caused by the device or the patient.

### 2.2. Acquisition and Evaluation of the CBCT Images

The scans were performed using the NewTom 5G CBCT device (Quantitative Radiology, Verona, Italy). The scans were conducted at 110 kVp and 1–20 mA and with field-of-view (FOV) sizes of 15 × 12 cm, 12 × 8 cm, and 18 × 16 cm, with the voxel size parameters ranging from 0.2 mm to 0.3 mm.

### 2.3. The Ground Truth

**Figure 1:**
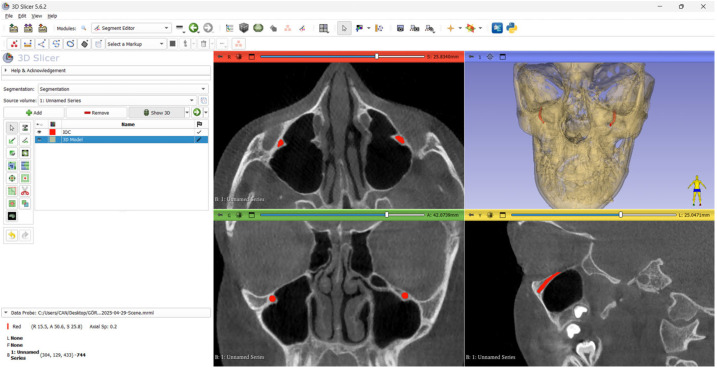
The right and left infraorbital canals were manually labeled using the 3D Slicer imaging software.

The CBCT images containing the IOC were converted into DICOM format and then sliced using the 3D Slicer imaging software (version 4.10.2; MIT, Cambridge, MA, USA) by an expert with 5 years of experience (Dentomaxillofacial Radiologist-1). After the manual labeling process was completed, the accuracy of the segmentation was verified by a more experienced expert (Dentomaxillofacial Radiologist-2). In cases where a consensus could not be reached, the opinion of a third expert (Dentomaxillofacial Radiologist-3) was sought. The manual segmentation process involved manually delineating the boundaries of the IOC using anatomical knowledge. Manual segmentation was performed using all three planes (sagittal, coronal, and axial), ensuring that the boundaries of the IOC were not exceeded and no gaps were left within the canal (Figure 1). The DICOM images in which manual segmentation had been completed were then converted into NIfTI format for further processing. The resulting NIfTI images were prepared for automatic segmentation using the nnU-Net v2 model.

### 2.4. Test Data

**Figure 2:**
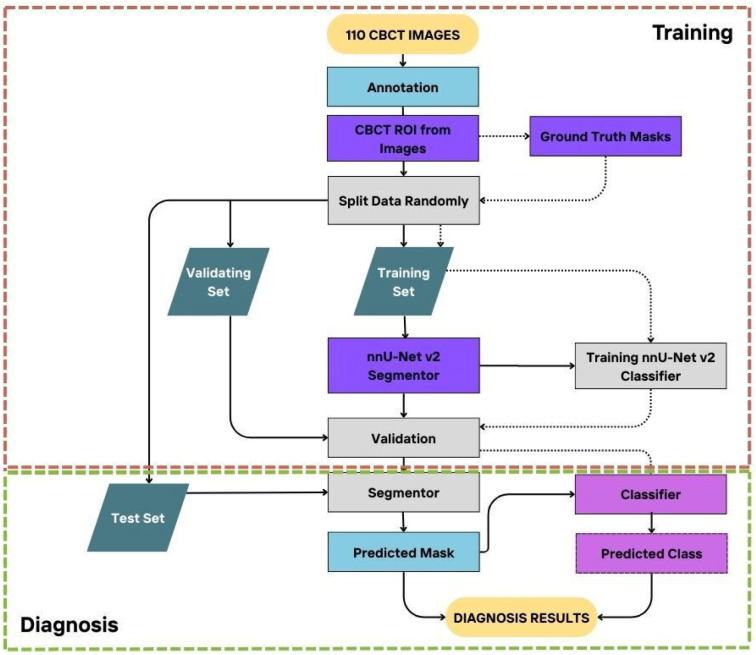
A workflow diagram including the data preparation, model training, and prediction stages of the nnU-Net v2 model.

In the present study, a dataset consisting of CBCT images from 110 patients was used. The data preparation and replication processes were successfully completed. A total of 220 IOCs from the right and left sides of each patient were included in this study. To enhance the accuracy and reliability of the dataset, a 10-fold cross-validation method was applied. Using this method, CBCT images from 88 training, 11 validation, and 11 test groups were utilized, allowing for a more robust evaluation of the model’s overall performance and success rate (Figure 2).

### 2.5. The Model

In this study, the nnU-Net v2 model, a 3-dimensional deep learning (DL) model developed for medical image segmentation, was used. The first version of nnU-Net demonstrated an outstanding performance in segmentation tasks and has become the standard in many medical imaging applications [22]. The nnU-Net v2 model has been made more flexible, faster, and more performant compared to its previous version, providing an automated solution to achieve the best results in medical image segmentation. When designing deep learning models, there are several parameters that determine the performance of the algorithms used in the model. These parameters are variables that can be chosen based on the problem, dataset, or system used. These variables are referred to as hyperparameters. The hyperparameters used in this study are provided in Table 1.

**Figure 3:**
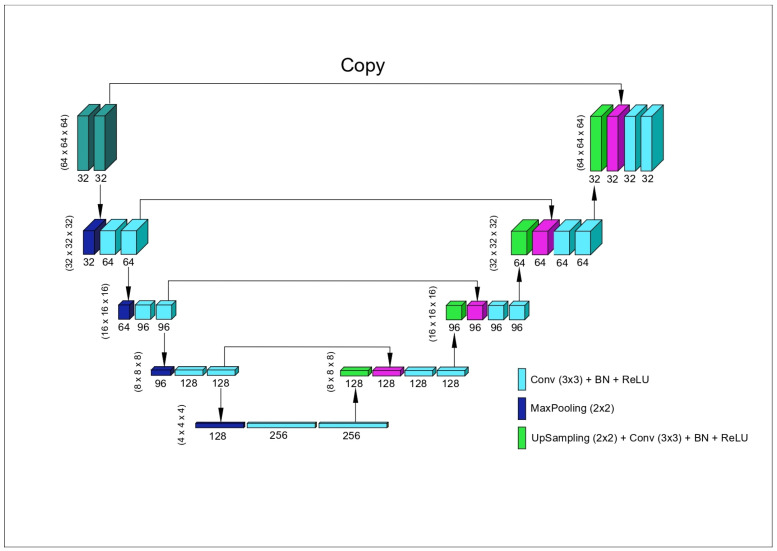
nnU-Net architecture: an automatically configured U-Net-based segmentation algorithm.

In this study, the PyTorch library (v3.6.1; Python Software Foundation, Wilmington, DE, USA) was used to build, train, and run the deep learning models in a Python environment. All data input to the model were standardized to a size of 64 × 64 × 64 (Figure 3). At this stage, preprocessing steps were applied to the data inputs to prepare them for model training. Additionally, data augmentation techniques were used and normalization processes were carried out to improve the model’s performance. The model training was performed for 1000 epochs on 88 CBCT images with a learning rate of 0.00001.

### 2.6. Evaluation

**Figure 4:**
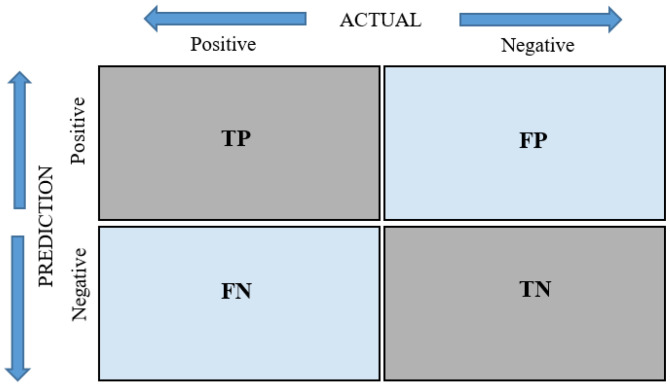
Confusion matrix.

In this study, the success and performance of the nnU-Net v2 model in segmenting the infraorbital canal in CBCT images were evaluated using several metrics. The model’s accuracy, precision, and recall values were calculated using a confusion matrix (Figure 4). Additionally, the overall success and performance of the model were measured using the Dice Score (DC), Intersection over the Union (IoU), F1-score, and 95% Hausdorff distance (95% HD) metrics. These values are obtained by calculating the number of pixels in the relevant regions by superimposing the result set obtained from semantic segmentation and the mask regions created by experts, which are taken as the ground-truth reference set. Additionally, the overall accuracy of the model was measured, and the area under the ROC curve was calculated (AUC). The goal was to comprehensively evaluate the model’s performance using these metrics. The formulas and values for the relevant parameters are shown in Table 2.

## 3. Results

**Figure 5:**
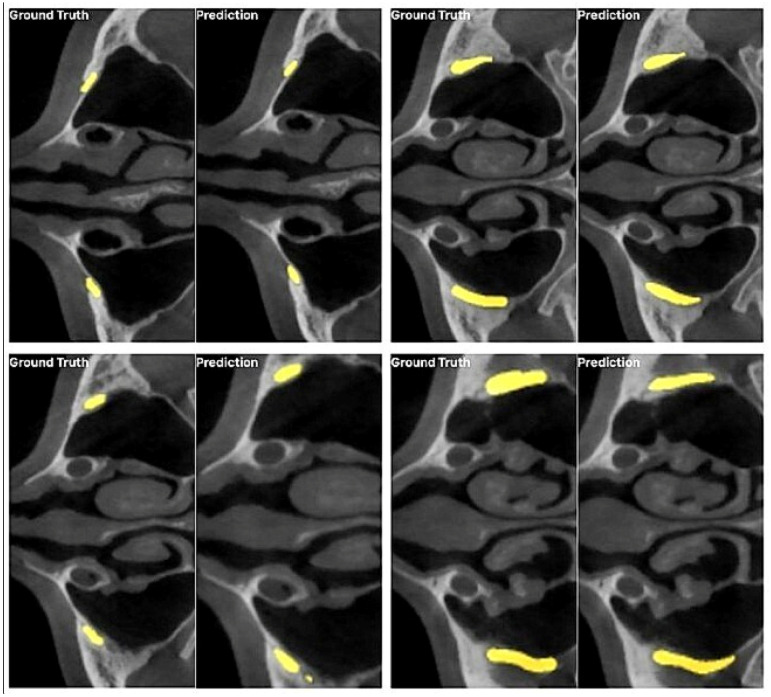
An axial section showing the ground truth for the infraorbital canal and images of the model’s prediction.

The predictive analysis of the proposed nnU-Net v2 model is shown in Figure 5. The precision, recall (sensitivity), and accuracy of the model are three key metrics used to evaluate its overall performance. Recall measures the model’s ability to correctly identify actual positive cases, while precision indicates how accurately the model classifies the predicted positive cases. In this study, the model’s precision, recall, and accuracy were calculated as 0.7479, 0.8231, and 0.9999, respectively. These results show that the model successfully made correct predictions 74.79% of the time and correctly classified the positive class 82.31% of the time (Table 2).

In the present study, the DC, F1-score, and IoU metrics were calculated to evaluate the model’s performance. The results obtained were an important indicator in determining the model’s segmentation success. The DC, with a value of 0.7792, indicated that the model overlapped with the true positive examples 77.92% of the time. The F1-score was calculated as 0.7837, showing that the balance between the accuracy and precision was good. The IoU value was calculated as 0.6402, indicating that the intersection ratio between the predicted area and the true area was 64.02%. The 95% Hausdorff distance was calculated as 0.7661, demonstrating that the predicted boundaries were largely in agreement with the actual values (Table 2). These metrics were used to evaluate the model’s overall accuracy, precision, and generalization ability, and the values obtained showed that the model demonstrated a successful performance.

**Figure 6:**
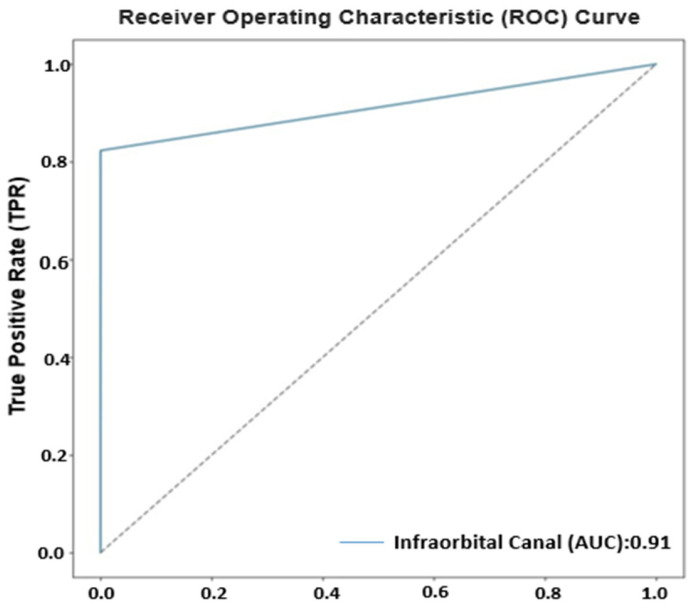
Visualization of the model’s ROC curve, the true positive area, and the AUC value.

The ROC curve is an important tool used to evaluate the performance of a classification model. The AUC value represents the area under this curve and indicates the model’s discriminatory power. A model with an AUC value of 0.91 demonstrates a good performance. This value indicates that the model is more successful than a system making random guesses (AUC = 0.5) and generally has a high accuracy rate. As the AUC value approaches 1, the model’s ability to distinguish between positive and negative classes increases. Therefore, a high AUC value like 0.91 shows that the model minimizes misclassifications and makes accurate predictions, providing reliable classification. The graph below shows the ROC curve and the AUC graph during the model’s training process (Figure 6).

**Figure 7:**
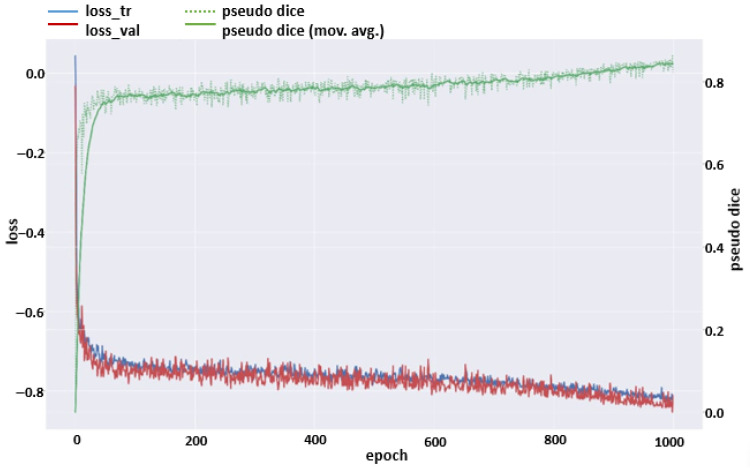
A curve showing the change in the loss function during the model’s training process.

A decreasing loss function curve indicates that the model is learning better over time and that the error rate is decreasing. The following loss function graph shows how the training and validation loss changed with respect to the number of epochs (Figure 7). The regular decrease in the training loss indicates that the model’s learning process is progressing successfully, while the similar trend in the validation loss suggests that the model has a good generalization capacity.

## 4. Discussion

In this study, automatic segmentation of the IOC in CBCT images was performed using the nnU-Net v2 model. The infraorbital nerve, which passes through the IOC, is responsible for sensory innervation of the face between the lower lid of the eye and the upper lip [5,23]. Maxillofacial surgeries, implant applications, and cosmetic procedures are usually performed under regional block anesthesia. Therefore, accurately determining the position of the IOC will help prevent complications due to surgical and anesthetic interventions [24,25].

An abnormal course or location of the IOC in the maxillary sinus or the orbital regions may lead to iatrogenic complications, potentially resulting in conditions such as hypoesthesia or paresthesia [6,24,26,27]. In trauma cases, especially facial and maxillary injuries, the anatomical location of the IOC should be thoroughly evaluated through radiological imaging [2,28]. Surgical intervention planning should be made in accordance with this assessment to preserve the integrity of the infraorbital nerve and vessels [27]. As a result, accurate determination of the IOC’s location is crucial in preventing unwanted complications in both diagnostic and treatment processes [26].

CBCT, with its high-resolution, three-dimensional imaging capability, allows for detailed examination of the IOC and the surrounding anatomical structures [4,13,14]. However, manual segmentation of CBCT images is a time-consuming, operator-dependent, and tedious process. Additionally, physicians who have not received sufficient CBCT training may struggle with evaluating the localization of anatomical structures [20,29,30,31]. To address these limitations, numerous studies in recent years have focused on automatic segmentation of anatomical structures using AI and DL algorithms [10,20,30,32,33].

Lahoudet al. [34] performed automatic segmentation of the mandibular canal using 235 CBCT images and an AI model, achieving a DC of 0.774 and an IoU value of 0.636, respectively. Their results indicate that the segmentation performance of the model is reasonably good. In the present study, the DC and IoU values were calculated as 0.7792 and 0.6402, respectively. These values indicate a slight improvement compared to Lahoud et al.’s [34] study. In particular, the increase in the DC suggests that the segmentation shows better alignment with the manual placements. Similarly, the increase in the IoU value indicates that the overall overlap performance is somewhat more successful.

Deniz et al. [30] performed automatic segmentation of the nasopalatine canal using the YOLOv5x-seg model with 200 CBCT images. In their study, the accuracy, precision, F1-score, and IoU parameters were evaluated, with values of 0.9680, 0.9953, 0.9815, and 0.9636 reported for the group without NPC furcation. However, in the present study, the corresponding metrics were found to be 0.9999, 0.7479, 0.7837, and 0.6402, respectively. The results obtained indicate that the segmentation accuracy of the model used by Deniz et al. [30] is higher. The main reasons for this difference may include factors such as the model architecture, the anatomical structure targeted, the distribution of the dataset, and the quality of the annotations. However, the metrics in this study also show an acceptable performance for the IOC, and optimizing the system with larger and more balanced datasets may offer potential improvements.

Previous studies involving the segmentation of the temporomandibular joint [10], the maxilla and mandible [33], and the maxillary sinus [35] have shown that these AI-based models performed better compared to that in this study. This can be explained by the fact that the anatomical structures in question are simpler and larger in size compared to the IOC. Therefore, the smaller and more complex anatomical structure of the IOC may make its visualization and segmentation more challenging compared to other structures. The anatomical structure of the IOC may have contributed to the relatively lower performance of the AI-based model used in this study compared to that demonstrated in other studies [10,33,35].

The nnU-Net v2 model has demonstrated a consistent segmentation performance despite the anatomical challenges of the infraorbital canal. The continuity of structures with the canal anatomy, variable sizes, and irregular boundaries have been identified as factors that frequently challenge segmentation algorithms [36]. However, this AI-based model was able to effectively trace the radiolucent space of the IOC and accurately delineate its boundaries. This is a significant achievement, especially considering the IOC’s highly variable and fine structural features. The model’s ability to visually track the fine details of the canal’s cortical structure and classify them correctly demonstrates the potential strength of AI-based segmentation systems in medical imaging.

Integrating the AI model used in this study, integrated into CBCT devices with user-friendly interfaces and algorithms, could make significant contributions to improving patient outcomes and optimizing procedure times in surgical and anesthesia procedures. This integration could also enable improvements in operational efficiency and diagnostic accuracy and personalized treatment approaches in the detection of different anatomical structures and conditions [37,38,39]. Thus, rapid and accurate detection of the IOC reduces the clinical workload and contributes to the prevention of potential complications in maxillary sinus surgery, implant applications, and cosmetic interventions.

There are some limitations in this study. The smaller diameter of and anatomical variations in the IOC introduce potential limitations that may have affected the model’s performance. Therefore, it could be improved further with more specific algorithms targeting challenging structures like the IOC. Additionally, the technical limitations of CBC images complicate this process. Noise in the images, low soft tissue contrast, and metal artifacts can hinder clear visualization of the radiolucent structure of the IOC. This makes it more difficult for the model to accurately delineate the segmentation boundaries. Finally, the use of a larger and more diverse dataset could improve the generalization capabilities of the models by learning different age groups, anatomical variations, and various imaging conditions.

## 5. Conclusions

This paper demonstrated the potential of the nnU-Net v2 model for automatic and reliable segmentation of the IOC in CBCT images. Accurate and automated segmentation of the IOC may provide significant advantages to clinicians in preoperative planning by reducing the risk of nerve injury during surgical interventions. These findings suggest that integrating AI-based segmentation tools into dental practice may help improve surgical planning and patient safety. Furthermore, future development of user-friendly interfaces and their integration into routine dental CBCT software may enhance the clinical applicability of such tools and facilitate their adoption in everyday workflows. Additionally, automatic segmentation may contribute to analysis and classification of the anatomical variations across different populations, supporting the development of more personalized and reliable treatment approaches. When applied to large datasets, this technology may also serve as an important resource for clinical research by providing valuable insight into the morphological characteristics of the IOC.

## Figures and Tables

**Table 1:** The details of the training parameters and the performance metrics of the proposed nnU-Net v2 model.

Parameter	Value
Model	nnU-Net v2
Epoch	1000
Batch Size	2
Learning Rate	0.00001
Optimization	ADAM
Activation	ReLU

**Table 2:** The metric values obtained from the fully automated deep-learning-based segmentation of the IOC in the CBCT images, along with their corresponding formulas, are presented below.

Metrics	Metric Formula	Metric Value
True Positive		17,288.7
False Positive		4043.5
False Negative		3073.1
Precision	TP/(TP + FP)	0.7479
Recall (Sensitivity)	TP/(TP + FN)	0.8231
Accuracy	(TP + TN)/(TP + TN+ FP + FN)	0.9999
Dice Score (DC)	(2 × TP)/(2 × TP + FP + FN)	0.7792
Intersection over the Union (IoU)	(|A∩B|)/(|A∪B|)	0.6402
F1-Score	2 × (Precision × Recall)/(Precision + Recall)	0.7837
95% Hausdorff Distance (95% HD) mm	*d*_H95_(A, B) = max(*d*_95_(A, B), *d*_95_(A, B))	0.7661

Notes: TP: True Positive, FP: False Positive, FN: False Negative.

## Data Availability

The original contributions presented in this study are included in the article. Further inquiries can be directed to the corresponding author.
